# Patient Digital Engagement With After Visit Summary in Ambulatory Care

**DOI:** 10.1001/jamanetworkopen.2026.15020

**Published:** 2026-05-28

**Authors:** Ryan Thomas Halvorson, Sophia Wang, Liam Wong, Akash Vasanthan, Robert Thombley, Brian T. Feeley, A. Jay Holmgren

**Affiliations:** 1 Department of Orthopaedic Surgery, University of California San Francisco, San Francisco; 2 Department of Medicine, University of California San Francisco, San Francisco

## Abstract

**Importance:**

The after visit summary (AVS) is believed to be a critical mechanism for postvisit communication, and health systems are widely adopting digital AVS systems to comply with federal regulations. However, little is known regarding the physician time cost of writing the AVS and whether patients routinely engage with the digital AVS.

**Objective:**

To assess patterns of patient digital AVS engagement following ambulatory care visits and the associated physician time cost.

**Design, Setting, and Participants:**

This cross-sectional study analyzed ambulatory care visits that occurred between June 1, 2018, and May 31, 2023, at a large, urban, academic health care system with multiple hospitals and outpatient clinics. All ambulatory visits by adults between the study period that were captured in the institutional electronic health record system were included. Data were analyzed in May 2025.

**Main Outcomes and Measures:**

The primary outcome was engagement with the digital AVS through the patient portal following an ambulatory visit. Secondary measures included patient demographic factors, visit variables (including clinic specialty [primary care, urgent care, medical specialties, surgical specialties] and visit type [video, nonvideo]), and physician-specific behaviors (including time spent writing patient instructions, using composition tools, and physically printing the AVS).

**Results:**

A total of 6 262 623 ambulatory care visits were analyzed. Most visits were made by female patients (3 890 100 [62.1%]), and 51.0% of all patients were aged 55 years or older. Digital AVS engagement increased from 20.8% in 2018 to 37.6% in 2023. Physicians spent a median (IQR) duration of 1.38 (0.52-3.28) minutes per visit writing patient instructions, despite the AVS not being viewed in 62.4% of encounters. AVS composition varied by physician specialty, with surgeons using a greater percentage of templated text (63.6% of AVS text) and medical specialists entering more text manually (50.7% of AVS text). Patient engagement varied by demographic factors, with higher engagement among married vs single patients (AME, 6.6 [95% CI, 6.1-7.1] percentage points) and retired vs employed patients (AME, 5.1 [95% CI, 4.5-5.6] percentage points). Lower engagement with the digital AVS was found among males vs females (AME, –3.1 [95% CI, –3.7 to –2.5] percentage points), non-English speakers vs English speakers (AME, –15.0 [95% CI, –15.9 to –14.2] percentage points), and those with Medicare (AME, –3.3 [95% CI, –4.2 to –2.5] percentage points) or public insurance (AME, –3.6 [95% CI, –4.4 to –2.9] percentage points) compared with private insurance. Physician behavior was also associated with digital AVS engagement, with presence vs nonpresence of physician-written patient instructions associated with higher engagement (40.5% vs 34.3%) and printed vs nonprinted AVS associated with lower engagement (31.6% vs 38.8%). Compared with primary care, medical and surgical specialties were associated with lower digital AVS engagement (medical: AME, –4.3 [95% CI, –5.3 to –3.2] percentage points; surgical: AME, –2.9 [95% CI, –4.1 to –1.7] percentage points), while urgent care showed no significant difference (AME, –0.6 [95% CI, –2.2 to +1.0] percentage points).

**Conclusions and Relevance:**

In this cross-sectional study of ambulatory visits, digital AVS engagement increased but remained low and was associated with a high time investment for physicians. This inefficiency in postvisit communication emphasizes the need to reconsider such communication, particularly with unmarried males, non-English-speaking populations, and publicly insured populations, and in specialty care settings.

## Key Points

**Question:**

What factors are associated with patient engagement with the digital after visit summary (AVS) following an ambulatory care visit, and what is the time cost to physicians?

**Findings:**

In this cross-sectional study of 6 262 623 ambulatory care visits, digital AVS engagement increased from 20.8% in 2018 to 37.6% in 2023 and was more likely when physicians included personalized patient instructions and among married, female, and retired English-speaking patients in the primary care and urgent care settings. Physicians spent a median time of 1.38 minutes per visit authoring instructions, with differences according to composition strategy and specialty.

**Meaning:**

The study finding that digital AVS engagement increased over time but remained low highlights a critical inefficiency in postvisit communication.

## Teaser

This cross-sectional study evaluates patterns of patient engagement with the digital after visit summary (AVS) following an ambulatory care visit and assesses physician time burden associated with the AVS.

## Introduction

Originally required by the Centers for Medicare & Medicaid Services Merit-Based Incentive Payment System,^1^ the practice of providing patients with an after visit summary (AVS) has become standard in US health care. The AVS, which consists of a personalized overview of the diagnosis, management plan, and follow-up plan, is believed to be a critical patient communication mechanism in ambulatory care.^2^ Prior studies of the AVS and its predecessors have demonstrated patient satisfaction and information retention benefits, particularly when information is accurate, relevant, and understandable.^2,3,4^ While historically the AVS was printed at the conclusion of visits, many health systems have transitioned to digital AVS delivery through patient portals integrated in the electronic health record (EHR) to reduce paper waste and to comply with regulations of the Centers for Medicare & Medicaid Services, which mandates providing patients with electronic access to their health information.^1,3^ This transition has been expedited by increased digital health care delivery following the COVID-19 pandemic,^5^ including telemedicine visits for which printed AVS is impossible. Despite widespread implementation of the digital AVS, rates of patient engagement are unknown, and the physician burden associated with generating the AVS has not been quantified.

Patient factors associated with engagement with the digital AVS are not well known. A pre–COVID-19 survey found that, while 76% of patients were aware of the AVS being available through the online patient portal, only 54% reported accessing it.^6^ Patients who accessed the AVS tended to be married and less educated, but the survey only achieved a response rate of 23%, and AVS engagement was self-reported. Actual administrative and EHR data are needed to identify more precise statistics regarding engagement. Other research on the content and quality of AVS documentation has suggested that patients find the AVS useful, especially with personalized instructions.^2^ Furthermore, the dynamics of access have likely changed over time, especially given that the COVID-19 pandemic dramatically increased patient portal enrollment and shifted the demographics of portal users.^7^ Identifying patient-, physician-, and system-level characteristics associated with AVS engagement may inform strategies to enhance its efficacy and promote access, such as better targeting outreach and educational initiatives toward patient groups who underuse the digital AVS.

For physicians, the time cost of AVS documentation has not been established, despite the well-documented time costs of other EHR tasks and association with burnout.^8,9^ Depending on their specialty, US physicians spend between 45 and 115 minutes per day on EHR tasks.^10^ Physician time spent on other patient communication tasks, such as patient messaging, has increased 57% per day compared with time spent before the COVID-19 pandemic and is associated with 2.3 minutes of increased EHR time per message.^11^ Given the modern practice environment with high levels of EHR burden, it is critical to assess the costs of generating the AVS on physician time to both facilitate a cost-benefit calculus and to help assess the potential value of interventions to reduce AVS documentation time, such as artificial intelligence (AI) ambient scribe tools that automatically generate the AVS.

Given the widespread implementation of the digital AVS, this study aimed to assess patterns of digital AVS engagement following ambulatory care visits and the associated physician time cost. Specifically, we sought answers to several key questions regarding utility and burden. First, what proportion of patients engage with the digital AVS? Second, what patient demographic, clinical, or physician-related factors are associated with AVS engagement? Third, what is the physician time cost of writing patient instructions within the AVS? Results from this study could inform health systems regarding when and where the AVS is an effective method of patient communication, which may help develop more efficient processes for allocating the time of physicians and other clinicians as well as target outreach, such as care coordinator telephone check-ins, to ensure that patient groups who may underuse the digital AVS are informed of any necessary follow-up care.

## Methods

### Setting and Sample

This retrospective cross-sectional analysis of all adult ambulatory care visits between June 1, 2018, and May 31, 2023, was conducted in May 2025 at the University of California San Francisco Medical Center. This large, urban, academic health care system comprises multiple hospitals and outpatient clinics and provides both primary and specialty care to a large, diverse population. All visits were captured within a single EHR system with standardized patient communication across sites. In accordance with 45 CFR §46.104, this cross-sectional study is exempt from ethics review and the informed consent requirement because all patient data used were completely deidentified. We followed the Strengthening the Reporting of Observational Studies in Epidemiology (STROBE) reporting guideline.

All adult ambulatory care visits were eligible for inclusion. Pediatric patients were excluded, as were hospital-based and emergency department visits because the patient communication workflows are different in these settings. The start date of June 2018 was selected as it corresponded with an update to the backend of our institutional EHR (Epic Clarity; Epic Systems), which enabled tracking of patient digital AVS engagement. The end date of May 2023 was selected as it corresponded with the adoption of AI-based scribes with AVS generation across ambulatory clinics, which may have shifted how physicians draft the AVS and patient instructions. At our institution, where it is standard practice to distribute the AVS in the patient’s preferred language, occasionally there may be manually entered text or web-based content in the EHR that is language discordant. Of note, the AVS is a combination of physician-written instructions (when present) and automatically generated content, including current medications, diagnoses, follow-up appointments, and clinic contact information.

### Primary and Secondary Outcomes

The primary outcome was patient engagement with the digital AVS, defined as a patient accessing the digital AVS through the patient portal following an ambulatory visit. Engagement was identified using backend EHR activity logs that record AVS viewing events. A single AVS view at any time point following the encounter was counted as engagement.

Secondary measures included patient demographic characteristics (age, sex, marital status, primary language, race and ethnicity, insurance status, and employment status), visit-level variables (clinic specialty, visit type [eg, new patient, follow-up, video visit, nonvideo visit], year), and physician-specific behaviors related to AVS documentation. Race and ethnicity were included in this analysis to characterize the sample and were not analyzed further. Physician behaviors included whether the physician authored patient instructions as part of the AVS and whether the AVS was printed during the visit. These variables were obtained directly from the EHR metadata and time-stamped activity logs associated with each visit.

The duration of time spent by physicians writing patient instructions was recorded from the EHR metadata as the duration of time the cursor was active within the instructions box. The length (in characters) of each set of patient instructions as well as the percentage of patient instruction text attributable to manual entry, copy-paste functionality, and templating (eg, Epic SmartPhrases) were also recorded.

### Statistical Analysis

Descriptive statistics for our patient and physician sample are reported along with rates of engagement with the printed or digital AVS over time. Descriptive statistics for time spent writing the AVS as well as the composition tools used to write the AVS are also reported.

The association between digital AVS engagement and demographic characteristics, visit characteristics, and physician behaviors were assessed using χ^2^ tests. A multivariable logistic regression model was generated to assess the association of patient, physician, and visit factors with AVS engagement. SEs were clustered 2 ways to account for patient and physician behavioral patterns using a 2-way cluster-robust variance estimator. Average marginal effects (AMEs) were calculated for each covariable and are presented as percentage point estimates and 95% CIs.

All analyses were carried out using R, version 4.2.2, and the multiwayvcov and margins packages (RStudio).^12^ The statistical significance threshold for the final model was set at 2-sided *P* < .05.

## Results

A total of 6 262 623 ambulatory care visits occurred between 2018 and 2023 and were included in the analysis (Table 1). Video visits comprised 13.5% of these encounters. Included visits were made by 3 890 100 females (62.1%) and 2 367 637 males (37.8%), of whom 51.0% were aged 55 years or older, 93.6% had documented English proficiency, and 52.9% were married. Among these patients, 16.5% were Asian, 4.9% were Black or African American, 11.4% were Latinx, 4.0% were multiracial or multiethnic, 0.2% were Native American or Alaska Native, 0.3% were Native Hawaiian or Other Pacific Islander, 1.0% were Southwest Asian and North African, and 56.4% were White individuals; smaller proportions of patients were grouped in the other and unknown categories. Additionally, 37.3% of patients were employed, 26.5% were retired, 17.7% were unemployed, and 16.9% had unknown employment status. Regarding insurance status, 38.9% of patients had private, 25.1% had Medicare, 9.9% had public, and 26.1% had unknown coverage.

**Table 1.** Patient Characteristics and After Visit Summary Engagement Rates (N = 6 262 623 Visits)

Characteristic	Patients, No. (%)			*P* value
	Total	Digital AVS engagement	No digital AVS engagement	
Physician-written instructions?				
No	3 719 127 (59.4)	1 274 699 (34.3)	2 444 428 (65.7)	<.001
Yes	2 543 496 (40.6)	1 030 490 (40.5)	1 513 006 (59.5)	
Year				
2018	543 891 (8.7)	113 189 (20.8)	430 702 (79.2)	<.001
2019	1 093 848 (17.5)	265 897 (24.3)	827 951 (75.7)	
2020	1 208 440 (19.3)	498 082 (41.2)	710 358 (58.8)	
2021	1 412 990 (22.6)	608 024 (43.0)	804 966 (57.0)	
2022	1 466 843 (23.4)	618 417 (42.2)	848 426 (57.8)	
2023	536 611 (8.6)	201 580 (37.6)	335 031 (62.4)	
Printed AVS?				
No	4 515 990 (72.1)	1 754 099 (38.8)	2 761 891 (61.2)	<.001
Yes	1 746 633 (27.9)	551 090 (31.6)	1 195 543 (68.4)	
Specialty				
Primary care	949 438 (15.2)	375 315 (39.5)	574 123 (60.5)	<.001
Medical specialties	2 680 741 (42.8)	975 381 (36.4)	1 705 360 (63.6)	
Surgical specialties	2 503 244 (40.0)	901 639 (36.0)	1 601 605 (64.0)	
Urgent care	129 200 (2.1)	52 854 (40.9)	76 346 (59.1)	
Visit type				
Nonvideo visit	5 417 363 (86.5)	1 929 087 (35.6)	3 488 276 (64.4)	<.001
Video visit	845 260 (13.5)	376 102 (44.5)	469 158 (55.5)	
Sex				
Female	3 890 100 (62.1)	1 468 234 (37.7)	2 421 866 (62.3)	<.001
Male	2 367 637 (37.8)	834 794 (35.3)	1 532 843 (64.7)	
Nonbinary	2131 (0.0)	989 (46.4)	1142 (53.6)	
Unknown	2755 (0.0)	1172 (42.5)	1583 (57.5)	
Primary language				
English	5 864 711 (93.6)	2 218 233 (37.8)	3 646 478 (62.2)	<.001
Other^a^	397 912 (6.4)	86 956 (21.9)	310 956 (78.1)	
Marital status				
Single	1 626 049 (26.0)	523 792 (32.2)	1 102 257 (67.8)	<.001
Divorced	390 958 (6.2)	144 061 (36.8)	246 897 (63.2)	
Married	3 317 575 (52.9)	1 333 315 (40.2)	1 984 260 (59.8)	
Widowed	375 526 (6.0)	128 123 (34.1)	247 403 (65.9)	
Other^b^	552 515 (8.8)	175 898 (31.8)	376 617 (68.2)	
Age, y				
18-34	1 185 453 (18.9)	429 572 (36.2)	755 881 (63.8)	<.001
35-54	1 880 260 (30.0)	681 437 (36.2)	1 198 823 (63.8)	
55-64	1 024 139 (16.4)	383 440 (37.4)	640 699 (62.6)	
65-74	1 193 732 (19.1)	473 406 (39.7)	720 326 (60.3)	
75-84	739 144 (11.8)	271 146 (36.7)	467 998 (63.3)	
≥85	239 895 (3.8)	66 188 (27.6)	173 707 (72.4)	
Insurance status				
Private	2 435 871 (38.9)	1 026 337 (42.1)	1 409 534 (57.9)	<.001
Medicare	1 570 527 (25.1)	594 805 (37.9)	975 722 (62.1)	
Public	621 368 (9.9)	191 882 (30.9)	429 486 (69.1)	
Unknown	1 634 857 (26.1)	492 165 (30.1)	1 142 692 (69.9)	
Employment status				
Employed	2 338 173 (37.3)	940 478 (40.2)	1 397 695 (59.8)	<.001
Retired	1 662 506 (26.5)	675 585 (40.6)	986 921 (59.4)	
Student	94 142 (1.5)	36 270 (38.5)	57 872 (61.5)	
Unemployed	1 110 189 (17.7)	365 327 (32.9)	744 862 (67.1)	
Unknown	1 057 613 (16.9)	287 529 (27.2)	770 084 (72.8)	
Race and ethnicity^c^				
Asian	1 032 596 (16.5)	411 313 (39.8)	621 283 (60.2)	<.001
Black or African American	306 831 (4.9)	80 916 (26.4)	225 915 (73.6)	
Latinx	716 654 (11.4)	228 561 (31.9)	488 093 (68.1)	
Multiracial or multiethnic	247 511 (4.0)	93 079 (37.6)	154 432 (62.4)	
Native American or Alaska Native	13 255 (0.2)	4236 (32.0)	9019 (68.0)	
Native Hawaiian or Other Pacific Islander	18 946 (0.3)	5435 (28.7)	13 511 (71.3)	
Southwest Asian and North African	62 975 (1.0)	23 644 (37.5)	39 331 (62.5)	
White	3 531 752 (56.4)	1 362 077 (38.6)	2 169 675 (61.4)	
Other^d^	182 844 (2.9)	57 059 (31.2)	125 785 (68.8)	
Unknown or declined	149 259 (2.4)	38 869 (26.0)	110 390 (74.0)	

Abbreviation: AVS, after visit summary.

^a^ Other languages included Afrikaans, Albanian, Algerian, Amharic, Arabic, Arabic (Middle Eastern), Arabic (Moroccan), Arabic (North African), Arabic (Yemeni), Aramaic, Armenian, Assyrian, Bengali, Bosnian, Bulgarian, Burmese, Cambodian, Catalan, Cebuano, Chinese (Cantonese), Chinese (Mandarin), Chinese (Mien), Chinese (Other), Chinese (Toishanese), Croatian, Czech, Dari, Dutch, Farsi, Flemish, French, French (African), French (Creole), French (Haitian), German, Greek, Gujarati, Haitian, Haitian (Creole), Hebrew, Hindi, Hmong, Hungarian, Igbo, Ilocano, Indonesian, Italian, Japanese, Karen, Khmer (Cambodian), Korean, Laotian, Lithuanian, Malayalam, Mam (Other), Mam (Todos Los Santos), Marathi, Mixteco, Mongolian, Mon-Khmer, Navajo, Nepali, Pashto, Persian, Philippine (Other), Polish, Portuguese, Portuguese (Brazilian), Portuguese (European), Punjabi, Romanian, Russian, Samoan, Serbian, Sign Language, Slovak, Somali, Swahili (Kiswahili), Swedish, Tagalog, Tamil, Telugu, Thai, Tibetan, Tigrinya, Tonga (Nyasa), Tonga Nyasa (Malawi), Tongan (Polynesia), Turkish, Ukrainian, Urdu (Indian), Urdu (Pakistan), Vietnamese, Yoruba, other, and unknown or declined.

^b^ Other marital status included legally separated (not divorced), dissolved, registered domestic partner, and significant other.

^c^ Race and ethnicity data were self-reported and obtained from the electronic health record.

^d^ Further definition not available.

**Figure 1.**
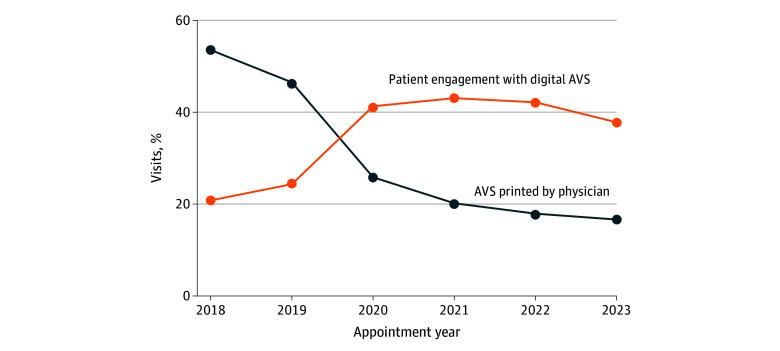
Line Graph of Patterns in Engagement With the Digital After Visit Summary (AVS) by Year for 6.3 million Visits

Over the study period, digital AVS engagement increased from 20.8% in 2018 to 37.6% in 2023 (Figure 1). In contrast, there was a decrease in printed AVS from 53.6% in 2018 to 16.7% in 2023. The AVS was not viewed in 62.4% of encounters.

### Univariable Analysis

Older patients were more likely to engage with the digital AVS, with engagement peaking at 39.7% for patients aged 65 to 74 years, before declining to 27.6% for patients 85 years or older (Table 1). Females engaged with the digital AVS more frequently than males (37.7% vs 35.3%; *P* < .001). Married patients demonstrated the highest engagement (40.2%), followed by divorced (36.8%) and widowed (34.1%) individuals, while single individuals (32.2%) and those with other relationship status (31.8%) were least likely to engage with their AVS (*P* < .001). English speakers engaged with their digital AVS more often than non-English speakers (37.8% vs 21.9%; *P* < .001). Among patients with a listed employment status, retired (40.6%) and employed (40.2%) individuals demonstrated the highest engagement, followed by students (38.5%), while unemployed patients (32.9%) and those with unknown status (27.2%) had lower engagement. Race and ethnicity demonstrated variability in engagement, with Asian (39.8%) and White (38.6%) patients showing the highest engagement compared with lower rates among Latinx (31.9%) and Black or African American patients (26.4%). Insurance status was also associated with engagement, with privately insured patients demonstrating the highest engagement (42.1%), followed by individuals with Medicare (37.9%), while those with public (30.9%) and unknown insurance status (30.1%) had lower engagement.

Regarding clinic specialty, 15.2% of visits were with primary care physicians, 42.8% with medical specialists, 40.0% with surgical specialists, and 2.1% with urgent care physicians. By specialty, urgent care physicians had the highest digital AVS engagement rate (40.9%), followed by primary care physicians (39.5%) and medical and surgical specialists (36.4% and 36.0%, respectively; *P* < .001). Engagement rates varied by visit type, with higher engagement following video visits compared with in-person visits (44.5% vs 35.6%; *P* < .001).

Physicians wrote instructions for 40.6% of visits, and the AVS was physically printed in 27.9% of visits, although this method decreased over the study period. Patients were more likely to engage with the digital AVS when physician-dictated instructions were present vs not present (40.5% vs 34.3%; *P* < .001). Visits for which the AVS was printed were associated with lower engagement compared with visits where the AVS was not printed (31.6% vs 38.8%; *P* < .001).

### Multivariable Analysis

**Figure 2.**
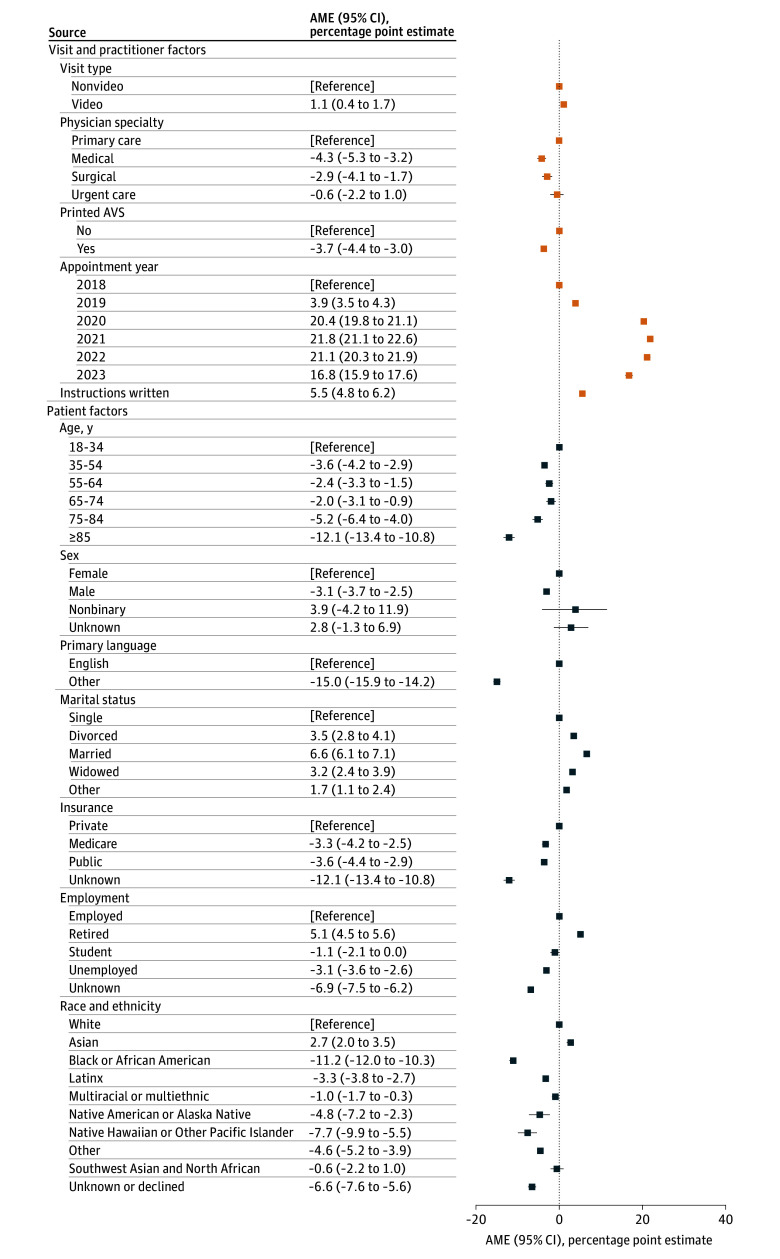
Dot Plot of Visit, Physician, and Patient Factors Associated With After Visit Summary (AVS) Engagement Results are presented from a multivariable logistic regression model with SEs clustered 2 ways to account for patient and physician behavioral patterns. Average marginal effects (AMEs) were calculated for each covariable and are presented as percentage point estimates and 95% CIs.

The presence of written patient instructions was associated with higher digital AVS engagement (AME, 5.5 [95% CI, 4.8-6.2] percentage points) (Figure 2), while AVS printing was associated with lower engagement (AME, –3.7 [95% CI, –4.4 to –3.0] percentage points). Engagement increased substantially after 2018, reaching a maximum in 2022 (AME, 21.1 [95% CI, 20.3-21.9] percentage points) and remaining elevated in 2023 (AME, 16.8 [95% CI, 15.9-17.6] percentage points).

Compared with primary care, medical and surgical specialties were associated with lower digital AVS engagement (medical: AME, –4.3 [95% CI, –5.3 to –3.2] percentage points; surgical: AME, –2.9 [95% CI, –4.1 to –1.7] percentage points), while urgent care showed no significant difference (AME, –0.6 [95% CI, –2.2 to +1.0] percentage points). Video visits were associated with slightly higher engagement compared with nonvideo visits (AME, 1.1 [95% CI, 0.4-1.7] percentage points).

Regarding patient-specific factors, non-English speakers were less likely to engage with the digital AVS compared with English speakers (AME, –15.0 [95% CI, –15.9 to –14.2] percentage points). Compared with single patients, married (AME, 6.6 [95% CI, 6.1-7.1] percentage points) and divorced patients (AME, 3.5 [95% CI, 2.8-4.1] percentage points) were more likely to engage, with smaller increases among widowed patients (AME, 3.2 [95% CI, 2.4-3.9] percentage points) and other groups (AME, 1.7 [95% CI, 1.1-2.4] percentage points). Males were less likely than females to engage (AME, –3.1 [95% CI, –3.7 to –2.5] percentage points), while those with nonbinary and unknown gender identity showed no significant differences.

Engagement with the digital AVS decreased with age. Compared with patients younger than 35 years, there was lower engagement among patients aged 35 to 54 years (AME, –3.6 [95% CI, –4.2 to –2.9] percentage points), 55 to 64 years (AME, –2.4 [95% CI, –3.3 to –1.5] percentage points), 65 to 74 years (AME, –2.0 [95% CI, –3.1 to –0.9] percentage points), and 75 to 84 years (AME, –5.2 [95% CI, –6.4 to –4.0] percentage points). The lowest engagement was observed in those 85 years or older (AME, –12.1 [95% CI, –13.4 to –10.8] percentage points).

Medicare, public, and unknown insurance were all associated with lower digital AVS engagement compared with private insurance (Medicare: AME, –3.3 [95% CI, –4.2 to –2.5] percentage points; public: AME, –3.6 [95% CI, –4.4 to –2.9] percentage points; unknown: AME, –12.1 [95% CI, –13.4 to –10.8] percentage points). Compared with employed patients, retired patients were more likely to engage (AME, 5.1 [95% CI, 4.5-5.6] percentage points), while unemployed patients were less likely to engage (AME, –3.1 [95% CI, –3.6 to –2.6] percentage points). Engagement by students was not significantly different. Compared with White patients, Asian patients had higher engagement (AME, 2.7 [95% CI, 2.0-3.5] percentage points), while Black (AME, –11.2 [95% CI, –12.0 to –10.3] percentage points) and Latinx patients (AME, –3.3 [95% CI, –3.8 to –2.7] percentage points) had lower engagement. Other racial and ethnic groups also demonstrated lower engagement, except for Southwest Asian and North African patients (AME, –0.6 [95% CI, –2.2 to +1.0] percentage points) whose engagement was not significantly different.

### Physician Behaviors

**Figure 3.**
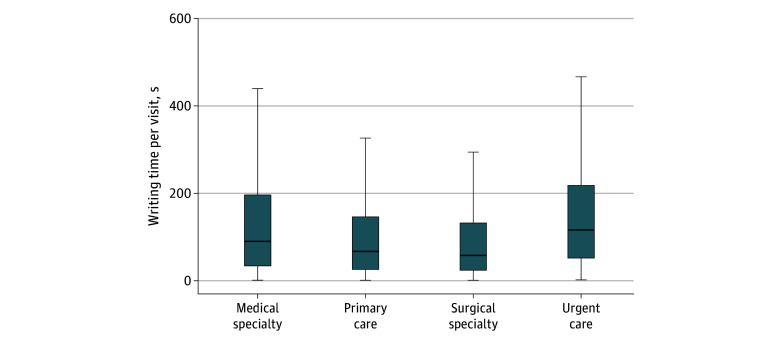
Box and Whisker Plot of Mean Time Spent by Physicians Writing Patient Instructions for the After Visit Summary The boxes represent the minimum and maximum time. The horizontal line inside boxes represents the median, and whiskers represent the IQR.

For visits with written patient instructions, physicians spent a median (IQR) duration of 1.38 (0.52-3.28) minutes per visit writing those instructions (Figure 3). Time spent writing patient instructions varied by specialty group. Urgent care physicians spent the most time on instructions, with a median (IQR) time of 1.93 (0.87-3.67) minutes per visit, followed by medical specialists (median [IQR] time, 1.53 [0.57-3.27] minutes per visit) and primary care physicians (median [IQR] time, 1.05 [0.43-2.22] minutes per visit). Surgical specialists spent the least amount of time writing instructions (median [IQR] time, 0.90 [0.37-1.93] minutes per visit).

There were also distinct patterns in the length and composition strategy of patient instructions by specialty (Table 2). Surgical specialists generated the longest instructions (mean [SD] length, 2659 [4443] characters per visit), with 63.6% of this composition derived from templates and only 26.9% written manually. Primary care physician instructions were also mostly generated from templates than written manually (55.1% vs 34.9%) but were shorter (mean [SD] length, 2234 [3468] characters per visit). Medical specialists produced the shortest instructions (mean [SD] length, 1340 [2417] characters per visit), which were composed of more manually written text (50.7%) compared with composition in any other group. Urgent care physician instructions had a mean (SD) length of 2543 (3496) characters per visit, with 40.1% written manually and 50.6% generated from templates.

**Table 2.** Physician Strategies for Writing Patient Instructions for the After Visit Summary

Specialty	Percentage of patient instructions		
	Derived from template	Written manually	Copied and pasted
Medical specialties	38.2	50.7	11.1
Primary care	55.1	34.9	9.9
Surgical specialties	63.6	26.9	9.5
Urgent care	50.6	40.1	9.3

## Discussion

Our cross-sectional analysis of 6 262 623 ambulatory visits demonstrated that, despite recent increases, digital AVS engagement remained low and was associated with a substantial physician time investment. Digital AVS engagement nearly doubled from 20.8% in 2018 to 37.6% in 2023. Despite the value of the AVS as a patient communication tool, patients never viewed it in 62.4% of encounters in 2023, with engagement varying across patient demographic characteristics, physician AVS behaviors, and visit type and clinic specialty. Physicians spent a median of 1.38 minutes per visit writing instructions and used a variety of composition strategies.

The upward trajectory in digital AVS engagement parallels the larger patterns in digital health engagement following the expansion of telemedicine during the COVID-19 pandemic.^13,14^ For example, Holmgren et al^11^ observed patient EHR messages increased by 57% per day compared with before the COVID-19 pandemic, suggesting increased patient familiarity with the patient portal. The adoption of telemedicine and video visits during this period also accelerated the digital transformation of communication modalities, as handing printed documents to virtual-visit patients became impractical. In the present study, patients were 9.0% more likely to engage with the digital AVS after video visits than in-person visits (44.5% vs 35.6%), suggesting greater familiarity with the patient portal. In our cohort, 93.3% of patients had patient portal access during the study period, reflecting the overall increase in digital adoption. Despite these increases in the use of digital AVS modalities as well as the lasting implications of COVID-19 for health care delivery, the most recent AVS engagement of only 37.6% underscores opportunities for improving postvisit communication.

When physicians wrote personalized instructions in the AVS, patients were significantly more likely to engage with the AVS. However, investing 1.38 minutes per visit in writing patient instructions is substantial for the physician, especially when the AVS is not viewed in 62.4% of encounters. For example, a physician who sees 32 patients in a clinic-day might spend 45 minutes (or roughly 3 standard patient visits per day) documenting AVS instructions. A physician with two 32-patient clinics per week and who works 48 weeks per year might spend 70.7 hours per year writing patient instructions. Given the association of these documentation tasks with physician burnout, identifying strategies to reduce time cost while maintaining quality of care and information transfer is critical.^8,9^ One possible method of maximizing efficiency with writing AVS instructions is to use a greater proportion of copied or templated text. In our analysis, AVS composition varied by subspecialty, with surgeons using a greater percentage of templated text (63.6% of patient instruction text) and medical specialists entering more text manually (50.7% of patient instruction text). Despite this difference, writing time investment remained high. Some institutions, including ours, have begun piloting AI scribes to create template clinic notes and patient instructions, which may be another viable strategy to reduce documentation burden while still providing personalized instructions to patients.

Although patients who were not given a printed AVS during an encounter were more likely to access the AVS digitally, this did not offset the engagement expected from having a physical AVS. As institutions push to eliminate printing and paper waste (only 27.9% of patients in this analysis had a printed AVS), it is important to monitor digital engagement to ensure overall engagement does not decrease.

The results of this study are consistent with prior surveys that demonstrate demographic differences in health care engagement.^2,6,15^ In our cross-sectional study, males were 2.4% less likely to engage with the AVS than females (37.7% vs 35.3%). Other studies have found that females are generally more proactive in health care communication and perceive health information as easier to understand, while younger males tend to use patient portals less frequently.^16,17^ We also observed a sharp decline in engagement among the oldest patients, which is consistent with prior studies demonstrating decreased patient portal access by older age groups.^16,18^ Barriers such as limited digital literacy and sensory or cognitive challenges often contribute to this gap across different ages.^18,19^ Patients in the 50- to 64-year age group demonstrated higher engagement, suggesting a substantial decrease with more advanced age. Interventions that have been recommended to improve patient portal access in this age group include one-on-one training, use of patient navigators, and simplification of user interfaces.^20^

Aside from English proficiency, marital status was associated with AVS utilization, with married patients 8.0% more likely to engage with the digital AVS compared with single patients (40.2% vs 32.2%). This finding aligns with prior studies linking marriage to greater outpatient care utilization and preventive care adherence.^21,22^ Patients without English proficiency had substantially lower rates of AVS engagement, corroborating prior work showing that health care information perceived as linguistically or culturally inaccessible reduces its usefulness.^23,24^ Despite the presence of translation services at our institution, patient portal registration and engagement require multiple other steps beyond the ambulatory care setting (eg, setting up a home internet connection, email address, and web browser). These requirements reinforce the need for a language-concordant patient portal to reduce communication barriers, especially as technology simplifies translation difficulty.^15,25,26^ Similar to differences in access based on language status, substantial disparities were observed in AVS access based on race and ethnicity and insurance status, particularly among Black or African American and Hispanic patients and those with public insurance. Prior research has reported racial disparities in overall patient portal access in cardiology and suggested targeted efforts to encourage patient portal signup in these specific groups.^27^ Although the present study did not explore specific underlying etiologies for racial and ethnic or socioeconomic differences, future studies could look specifically at possible contributing factors, including educational level and health care literacy.

AVS engagement also differed by visit type. Compared with the primary care setting, patients seen in medical and surgical subspecialties were significantly less likely to engage with the digital AVS, while patients visiting urgent care demonstrated the highest engagement. Prior studies have found that AVS distribution and quality are generally more standardized in primary care, although the actual content is variable.^4,28^ Less is known about AVS quality in procedural specialties, although some evidence suggests these summaries contain less information, potentially due to time constraints in these specialties.^4,29^ Observed between-specialty differences in AVS composition strategy may be due to differences in practice patterns, clinic time constraints, and relative complexity of patients seen in specific clinics. For example, a medical oncology clinic may be expected to have greater between-patient variability in postvisit communication than an orthopedic arthroplasty clinic, which is relatively standardized. There may also be AVS distribution strategies and reminders (ie, text messages, push notifications, and emails) that are not captured in this population-based analysis.

Future work could explore actual AVS content as well as how postvisit communication with at-risk groups might be optimized and how physician workflows might improve utilization. For example, interventions could target non-English-speaking populations through language-concordant instructions, older populations via simplified patient portal access, and unmarried males through alternative care delivery. Future studies could also assess the association between AVS engagement and clinical outcomes and treatment adherence. Finally, as AI-generated patient instructions become more prevalent, changes in AVS content and engagement should be monitored to assess changes in engagement, information quality, and physician time spent reviewing AI-generated content.

### Limitations

This study has several limitations. As a retrospective, descriptive analysis, the results are constrained by the quality of data available in the EHR. Although we could assess whether patients accessed the AVS digitally, we were unable to confirm whether patients actually read or understood the AVS or whether their engagement affected clinical outcomes. It is also possible that, in some cases, patients shared their username and password with their family or caregivers, which could artificially inflate patient viewership. However, it is not possible to delineate the frequency of this practice with the available data. Additionally, our analysis focused on quantitative patterns and did not evaluate causal associations or contextual factors that might explain observed differences. Similarly, although we could detect the presence of physician-generated patient instructions, we were not able to evaluate the actual text content of the written patient instructions to evaluate the quality of information.

## Conclusions

In this cross-sectional study of ambulatory visits, engagement with digital AVS increased but remained low among physicians and was associated with a high time investment for physicians. AVS engagement was more likely when physicians authored patient-specific instructions. Because the AVS has high value as a patient communication tool, these findings emphasize the need to reconsider postvisit communication, particularly with unmarried males, non-English-speaking populations, and publicly insured patients and in specialty care settings.

## Notes

Supplement.
Data Sharing Statement
