# Supplementary material for: Patient Digital Engagement With After Visit Summary in Ambulatory Care
Source: JAMA Netw Open. 2026 May 28;9(5):e2615020. doi: 10.1001/jamanetworkopen.2026.15020 (PMC13220110; doi:10.1001/jamanetworkopen.2026.15020)
Supplement: Supplement. — Data Sharing Statement [file jamanetwopen-e2615020-s001.pdf]

Data Sharing Statement

Halvorson. Physician Digital Engagement With After Visit Summary in Ambulatory Care. *JAMA Netw Open*. Published May 28, 2026. doi:10.1001/jamanetworkopen.2026.15020

Data
Data available: No

Additional Information
Explanation for why data not available: Deidentified data can potentially be made available upon request to the corresponding author after/if appropriate data sharing agreements can be arranged with the institution.
